# Not as simple as it seems: Extensive facility and training gaps in nursing home bathing

**DOI:** 10.1017/ash.2023.333

**Published:** 2023-09-29

**Authors:** Kristine Nguyen, Raveena Singh, Raheeb Saavedra, John Billimek, Steven Tam, Susan Huang

## Abstract

**Background:** Existing training for resident bathing in nursing homes (NHs) is brief and limited, likely because bathing is assumed to be intuitive. However, residents have complex skin issues, devices, dressings, and limited ability for self-care. We sought to assess bathing quality and to identify barriers to proper bathing techniques. **Methods:** We conducted a prospective observational study of bathing in 8 NHs in Orange County, California, involving a convenience sample of observed bed baths and showers conducted for quality improvement. NH staff were told that observation was occurring, and no feedback was given during or after bathing. Survey elements included cleansing of 6 specific body sites and adherence to bathing procedures (11 for bed baths and 17 for showers). Surveys also included queries to staff to further assess knowledge and perceived barriers. Observed lapses were documented, along with observer-determined reasons for noncompliance (ie, training issue, time pressure, facility issue (insufficient water temperature), resident refusal/behavior). Frequency of noncompliance with each element was tabulated for bed-baths and showers separately. Reasons for failure were displayed graphically. **Results:** In total, 50 bed baths (NH range, 5–8) and 50 showers (NH range, 4–7) were observed across 8 NHs. Lapses in bathing quality and process were extremely common for both bed baths and showers (Fig.). Inadequate body cleansing occurred for all observed body sites (88%–100% failure for bed baths, 58%–100% failure for showers). Most body areas were either skipped or sprayed with water without soaping. Procedural failures were high for both bed baths and showers (insufficient lather: 100% for bed bath and 40% for shower) lack of firm massage for cleaning (94% for bed bath and 90% for shower), failure to change wipes or cloths when dirty (100% for bed bath and 96% for shower), failure to follow clean-to-dirty sequence (100% for bed bath and 96% shower). In addition, failing to wrap or unwrap devices (73%) and failing to towel dry (94%) were common after showering. Reasons for failure were largely based on training or facility shortcomings (eg, insufficient hot water, inflexible showerhead attachment). Also, 86% of residents complained of being cold. Timing constraints and resident combativeness or refusal were rare. Staff-to-staff bathing advice most commonly involved competing for the “better shower” and “bathing early to get hot water.” **Conclusions:** Knowing how to appropriately bathe NH residents is not intuitive, and current training is brief and insufficient for high-quality resident care. Unacceptably high failures in proper bathing techniques in NHs necessitate re-evaluation of formal training and standardized practices to better cleanse residents. Moreover, common failures in facility processes for ensuring adequate water temperature and showerhead mobility for bathing or showering should be addressed.

**Figure:**
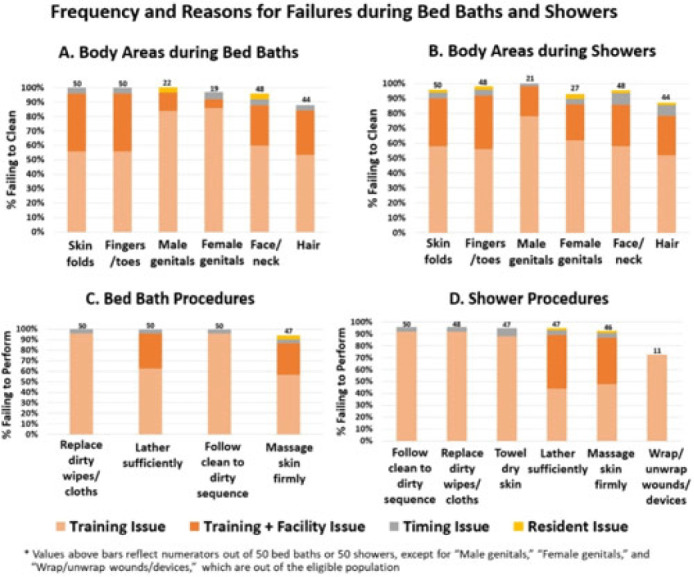

**Disclosures:** None

